# Tyrosine kinase inhibitor discontinuation in patients with chronic myeloid leukemia: a single-institution experience

**DOI:** 10.1186/s13045-018-0686-1

**Published:** 2019-01-03

**Authors:** Kamal Chamoun, Hagop Kantarjian, Rami Atallah, Graciela Nogueras Gonzalez, Ghayas C. Issa, Mary Beth Rios, Guillermo Garcia-Manero, Gautam Borthakur, Farhad Ravandi, Nitin Jain, Naval Daver, Marina Konopleva, Courtney D. DiNardo, Tapan Kadia, Naveen Pemmaraju, Elias Jabbour, Jorge Cortes

**Affiliations:** 1 Department of Leukemia, The University of Texas MD Anderson Cancer Center, Houston, TX USA; 2 Department of Biostatistics, The University of Texas MD Anderson Cancer Center, Houston, TX USA

**Keywords:** CML, TKI discontinuation, MR^4.5^

## Abstract

**Background:**

Patients with CML treated with TKI can have a life expectancy comparable to that of the general population. Due to the extended duration of TKI administration, treatment discontinuation has been increasingly sought.

**Methods:**

Medical records of 100 patients with CML who were in MR^4.5^ and discontinued their TKI outside clinical trials were reviewed.

**Results:**

After a median follow-up of 30 months (range, 5–112 months) after discontinuation, 35% and 17% lost MR^4.5^ and major molecular response (MMR), respectively. Only six patients lost MMR 12 months or more after discontinuation. Loss of MR^4.5^ was observed in 29% and 7% of patients with sustained MR^4.5^ duration of more than 2 and 6 years before discontinuation, respectively. By univariate analysis, there was a higher risk of loss of MR^4.5^ for patients who were treated for less than 87 months, received second or subsequent line TKI, never received interferon, or those with sustained MR^4.5^ for less than 6 years. By multivariate analysis, sustained MR^4.5^ for 6 years or more was the only significant predictor for durable response. Overall, 30% of patients who discontinued while in MR^4.5^ were retreated with 93% regaining MR^4.5^ at a median of 5 months.

**Conclusion:**

These results demonstrate that under proper conditions, treatment discontinuation is feasible outside of clinical trial setting. MR^4.5^ duration of 6 years or more before discontinuation is associated with very low risk of loss of MR^4.5^.

## Introduction

The introduction of imatinib, a selective tyrosine kinase inhibitor (TKI), revolutionized the treatment of chronic myeloid leukemia (CML) and changed the natural history of the disease [1, 2]. Subsequently, four additional TKIs were approved for the treatment of CML: bosutinib, dasatinib, nilotinib, and ponatinib. Second-generation TKIs, which have higher affinity for BCR-ABL fusion protein, were found to yield earlier and deeper responses when used in frontline compared to imatinib [3–5]. Although achievement of a complete cytogenetic response (CCyR) correlates with improved overall survival, better long-term event-free survival can be achieved with achievement of deeper responses (e.g., major molecular response (MMR) or MR^4.5^) as assessed by polymerase chain reaction (PCR) for BCR-ABL [6]. Patients with sustained deep molecular responses achieved more frequently with second-generation TKIs, may be considered for treatment discontinuation [3, 4, 7]. Currently, the National Comprehensive Cancer Network (NCCN) recommend discontinuation for patients in stable molecular response (BCR-ABL1 ≤ 0.01% IS) for ≥ 2 years [8]. Early responses, including BCR-ABL1 ≤ 10% at 3 months, ≤ 1% at 6 months, ≤ 0.1% at 1 year, and ≤ 0.01% later on, have been associated with higher probability of eventually having successful treatment-free remission [9]. Although TKIs are well tolerated compared to the majority of antineoplastic drugs, this chronic therapy may impact patient’s lives by either its associated adverse events (such as peripheral edema and muscle cramps with imatinib, pleural effusions with dasatinib, and vascular events with most of them), its influence on real-life events (e.g. pregnancy), or the financial burden to patients and society [4, 10–14]. Therefore, treatment discontinuation has been increasingly sought. Available data from prospective studies describing imatinib cessation in patients maintaining undetectable levels of BCR-ABL for at least 2 years shows a relapse rate of 36–61% [15–18]. Ongoing studies are evaluating TKI discontinuation in patients with variable durations of sustained MR^4.5^, with different levels of response, or in patients receiving second-generation TKIs [19–24]. Treatment discontinuation has been recommended mostly in the setting of clinical trials or in settings that provide optimal monitoring of patients to minimize the risk to patients [25]. The aim of this study is to evaluate the outcome of patients who stopped their TKI treatment at our institution outside a clinical trial. To the best of our knowledge, this represents the first experience with treatment discontinuation in clinical practice reported from the USA and the largest single-institution report on TKI treatment discontinuation to date.

## Patients and methods

Medical records of all patients with CML treated with TKI at MD Anderson Cancer Center and who subsequently discontinued therapy between 2003 and 2017 and had at least 5 months of follow-up were reviewed. This research was approved by the institutional review board (IRB), and a waiver for informed consent was obtained. We performed our analysis on 100 patients with MR^4.5^ at the time of treatment discontinuation, and we excluded patients with MR^4^ or MMR at the time of treatment discontinuation. Response criteria were defined as previously described [26]. Briefly, major molecular response (MMR) was defined as ≤ 0.1% BCR-ABL/ABL ratio in the international scale (IS), MR^4^ as a ratio of ≤ 0.01% IS and MR^4.5^ as a ratio of ≤ 0.0032% IS by quantitative real-time polymerase chain reaction (qPCR).

### Statistical analysis

The Kaplan Meier product limit method (Kaplan and Meier, 1958) was used to estimate the median time to event. Univariate Cox proportional hazards regression was used to identify any association with each of the variables and time to event outcomes. For each factor, medians, hazard ratios (HR), their 95% confidence intervals (CI), and proportional hazards regression *p* values were calculated. Classification and regression tree (CART) analysis using the martingale residuals of the Cox models were used to determine the functional form of continuous variables (i.e., times) to be added in the multivariate analyses. Statistical analysis was performed using Stata/SE version 15.1 statistical software (Stata Corp. LP, College Station, TX).

## Results

Between July 2003 and January 2017, 100 patients with CML chronic phase who were in MR^4.5^ discontinued TKI. Patients’ characteristics are summarized in Table 1. Sixty eight (68%) had received TKI as initial therapy for CML while 32 (32%) had been previously treated with interferon. In addition, 32% had received more than one TKI (27 had received 2 TKI and 5 had received 3 TKI) prior to discontinuation; of them, 25 (78%) had changed treatment due to intolerance, 6 (19%) due to resistance, and 1 (3%) due to study closure (ponatinib). The median time from diagnosis to treatment discontinuation was 119 months (range, 11–403). The median duration of TKI therapy was 108 months (range, 11–203), and the median time on the TKI received immediate prior to TKI discontinuation was 82 months (range, 1–198).

**Table 1:** Patients characteristics

Parameter	Median [range] or *N* (%) Total *N* = 100
Age (years)	49 years [16–74]
Female	57 (57)
Transcript type	
b3a2	50 (50)
b2a2	30 (30)
b2a2 + b3a2	17 (17)
b2a3	1 (1)
Sokal score	
Low risk < 0.8	35 (35)
Intermediate risk 0.8–1.2	21 (21)
High risk > 1.2	4 (4)
Not available	40 (40)
Treatment	
Previously treated with interferon	32 (32)
Frontline imatinib	43 (43)
Frontline dasatinib	13 (13)
Frontline nilotinib	9 (9)
Frontline ponatinib	2 (2)
Frontline bosutinib	1 (1)
Number of TKI lines	
1 line	68 (68)
≥ 2 lines	32 (32)
TKI at time of discontinuation	
Imatinib	47 (47)
Dasatinib	35 (35)
Nilotinib	14 (14)
Bosutinib	2 (2)
Ponatinib	2 (2)
Response	
BCR-ABL/ABL at 3 months	
< 10% (IS)	53 (95)
≥ 10% (IS)	3 (5)
Duration (months) from start of TKI to CCyR	4 [2–78]
Duration (months) from start of TKI to MMR	7 [1–91]
Duration (months) from start of TKI to MR^4.5^	17 [2–146]
At discontinuation	
Duration (months) from diagnosis to discontinuation	119 [11–403]
Duration (months) of last TKI before discontinuation	82 [1–198]
Imatinib	121 [11–198]
Dasatinib	64 [5–163]
Nilotinib	71 [1–119]
Bosutinib	47 [38–126]
Ponatinib	15 [15–15]
Duration (months) of MR^4.5^ before discontinuation	75 [1–178]
< 2 years	11 (11)
2 to 4 years	15 (15)
4 to 6 years	20 (20)
6 to 8 years	17 (17)
8 to 10 years	20 (20)
≥10 years	17 (17)
Reason for TKI Discontinuation	
Adverse events	53 (53)
Second cancer	2 (4)
Elective	47 (47)
Sustained MR^4.5^	38 (84)
Pregnancy	6 (13)
Financial reasons	3 (7)
Outcome after TKI discontinuation	
Follow-up (months) after TKI discontinuation	30 [5–112]
Loss of MR^4.5^	35 (35)
Loss of MMR	17 (17)
Retreatment	30 (30)
MMR after retreatment	29 (97)
MR^4.5^ after retreatment	28 (93)
Lost to follow-up after retreatment	1 (3)
Status on last follow-up	
CCyR	2 (2)
MMR	5 (5)
MR^4.5^	85 (85)
Death	8 (8)^a^

*CCyR* complete cytogenetic response, *IS* international score, *MMR* major molecular response, *MR*^*4.5*^ molecular response of 4.5-log reduction from baseline, *TKI* tyrosine kinase inhibitor

^a^All patients died while in MR^4.5^

Patients had achieved CCyR and MMR after a median of 4 months (range, 2–80) and 8 months (range, 1–132), respectively. MR^4.5^ was achieved after a median of 17 months (range, 2–146) on TKI. The median MR^4.5^ duration before discontinuation was 75 months (range, 1–178 months); 89 (89%) of them had a sustained MR^4.5^ for at least 2 years, and 54 (54%) for at least 6 years.

Fifty three (53%) discontinued therapy due to adverse events and 47 (47%) discontinued electively either due to sustained MR^4.5^ (*n* = 38), pregnancy (*n* = 6), or financial burden (*n* = 3).

### Outcome after treatment discontinuation

**Fig. 1:**
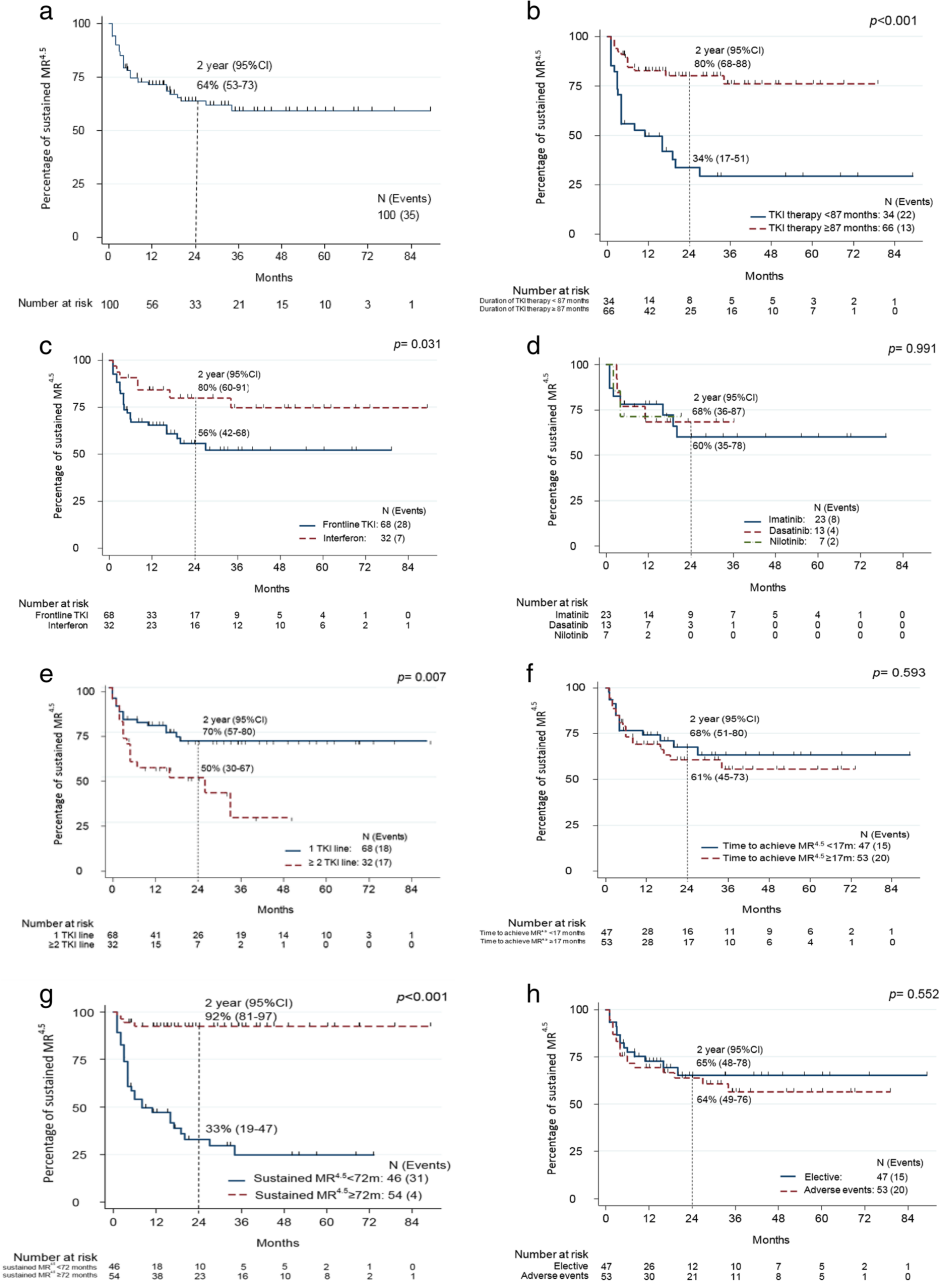
Kaplan Meier curve of percentage of sustained MR^4.5^ after discontinuation. **a** In the overall population, 2-year estimate: 64%, 95% CI [53, 73]. **b** According to duration of TKI treatment (< 87 months vs ≥ 87 months). **c** According to type of frontline treatment (TKI vs interferon). **d** According to type of frontline and only TKI (imatinib vs dasatinib vs nilotinib). **e** According to lines of treatment (first line vs two or more line). **f** According to duration to achieve MR^4.5^ (< 17 months vs ≥ 17 months). **g** According to sustained MR^4.5^ duration before discontinuation (< 72 months vs ≥ 72 months). **h** According to reason of discontinuation (elective vs adverse events)

**Fig. 2:**
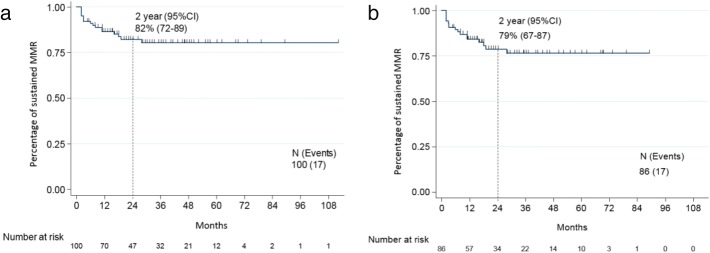
Kaplan Meier curve of percentage of sustained MMR after discontinuation. **a** In the overall population, two-year estimate: 82%, 95% CI [72, 89]. **b** Excluding patients who resumed therapy before losing MMR, 2-year estimate: 79%, 95% CI [67, 87]

After a median follow-up of 30 months (range, 5–112) following treatment discontinuation, a total of 35 (35%) patients lost MR^4.5^ (Fig. 1a) including 17 (17%) who lost MMR (Fig. 2). The median time to loss of MR^4.5^ was 4 months (range 1–34), and median time to loss of MMR was 4.5 months (range 2–19). Six (6%) of the patients who lost MR^4.5^ did so after 12 months from discontinuation (range, 16–34) (two of them lost MMR). Two patients who lost MR^4.5^ in less than 12 months (1 and 11 months) lost MMR at 18 and 28 months, respectively. Out of 35 patients with available data, three had additional clonal chromosome abnormalities in Philadelphia negative metaphases that were detected during their treatment period (range 3–18 months from start of treatment); two patients lost MR^4.5^ with one also losing MMR after TKI discontinuation.

### Recommencement of treatment

**Fig. 3:**
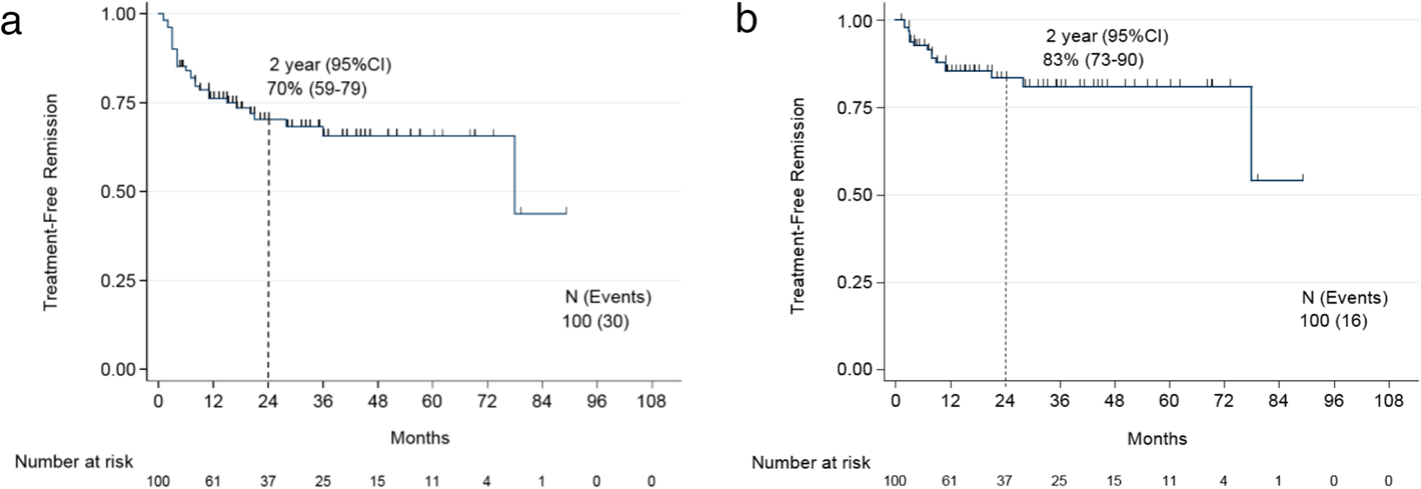
Kaplan Meier curve of treatment-free remission. **a** In the overall population, treatment-free remission at 2 years: 70%, 95% CI [59, 79]. **b** Censoring patients who resumed therapy before losing MMR, treatment-free remission at 2 years: 83%, 95% CI [73, 90]

Thirty (30%) patients resumed therapy (Fig. 3). These included 16 patients who lost MMR, 12 who lost MR^4.5^ but not MMR at the time treatment was resumed, and 2 who resumed therapy while still in MR^4.5^ (1 due to withdrawal syndrome, and 1 due to patient concern). Thus, the treatment-free remission, considering resumption of therapy as an event was 70% at 2 years; it is 83% when censoring patients who resumed treatment before losing MMR. Corresponding rates at 3 years were 68% and 81% and at 4 years 66% and 81%, respectively. Median follow-up after recommencement of treatment was 29 months (range 0–111). Out of 28 patients who were retreated for losing response, 26 (93%) have regained MR^4.5^, 1 patient regained MMR (12 months follow-up after resuming therapy), and 1 patient in CCyR was lost to follow-up after resuming therapy. Of the seven patients that did not resume therapy, two patients spontaneously regained MR^4.5^, four patients have maintained MMR after a median follow-up of 26 months (range, 5–40), and 1 patient who lost MMR remained in CCyR after 44 months of follow-up. The median time to regain MR^4.5^ was 5 months (range 2–18) after resumption of therapy; the median time to regain MMR was 3 months (range, 1–9).

At last follow-up, 92 patients were still alive in MR^4.5^ (*n* = 85), MMR (*n* = 5), or CCyR (*n* = 2), and 8 patients died while in MR^4.5^ due to leg gangrene post septic arthritis (1), myelodysplastic syndrome (1), or unknown causes (6).

### Factors associated with loss of MR^4.5^

In univariate analysis, duration of TKI therapy for 87 months or more was associated with the lowest risk of loss of MR^4.5^ after discontinuation compared to treatment duration of less than 87 months (20% vs 65%, respectively; *p* < 0.001) (Fig. 1b). Patients who had previously received interferon also had lower rate of MR^4.5^ loss than patients who never received interferon (22% vs 41%, respectively; *p* = 0.03) (Fig. 1c). Among patients who were still on their frontline TKI at the time of discontinuation, there was no significant difference in loss of MR^4.5^ for patients who received frontline imatinib, dasatinib, or nilotinib (35%, 31%, 29%, respectively; *p* = 0.99) (Fig. 1d). Patients who had only received 1 TKI had a significantly lower probability of loss of MR^4.5^ compared to those who had received two or more TKI by the time they discontinued therapy (26% vs 53%, respectively; *p* = 0.007) (Fig. 1e). Patients receiving imatinib, dasatinib, or nilotinib as their last TKI before discontinuation lost MR^4.5^ at a rate of 23%, 46%, and 43%, respectively (*p* = 0.16).

Median time to achieve MR^4.5^ was 17 months. There was no difference in the rate of loss of MR^4.5^ between patients who achieved this response in less than 17 months or in more than 17 months (32% and 38%, respectively; *p* = 0.6) (Fig. 1f). BCR-ABL/ABL ratio < 10% IS at 3 months had occurred in 53 patients and ≥ 10% in 3 patients. Their rates of loss of MR^4.5^ were 34% and 67%, respectively (*p* = 0.213) (Table 2).

**Table 2:** Clinical factors associated with the probability of loss of MR^4.5^

Variables	No. of pts	No. of events	Univariate Analysis	Multivariate analysis		
			*P*	HR	95% CI	*P*
Age						
< 65 years	87	30				
≥ 65 years	13	5	0.778			
Sex						
Male	43	18				
Female	57	17	0.160			
First treatment						
Interferon	32	7				
TKI	68	28	0.031	0.60	0.25 to 1.44	0.249
Frontline TKI						
Imatinib	43	19				
Dasatinib	13	4	0.463			
Nilotinib	9	2	0.439			
Bosutinib	1	1	0.210			
Poantinib	2	2	0.100			
Last TKI before discontinuation						
Imatinib	47	11				
Dasatinib	35	16	0.021			
Nilotinib	14	6	0.064			
Bosutinib	2	1	0.604			
Ponatinib	2	1	0.548			
Frontline and only TKI						
Imatinib	23	8				
Dasatinib	13	4	0.892			
Nilotinib	7	2	0.970			
Line of TKI						
1 line	68	18				
> 2 line	32	17	0.007	1.43	0.64 to 3.19	0.377
Response at 3 months						
BCR-ABL/ABL < 10% (IS)	53	18				
BCR-ABL/ABL ≥ 10% (IS)	3	2	0.213			
Transcript type						
b3a2	50	17				
b2a2	30	10	0.866			
b3a2 + b2a2	17	7	0.511			
Sokal score						
Low	35	10				
Intermediate	21	9	0.261			
High	4	0	0.999			
Reason for discontinuation						
Elective	47	15				
Adverse effects	53	20	0.557			
Duration to achieve MR^4.5^						
< 17 months	47	15				
≥ 17 months	53	20	0.593			
Duration of MR^4.5^						
< 72 months	46	31				
≥ 72 months	54	4	< 0.001	0.11	0.03 to 0.41	0.001
Duration of TKI therapy						
< 87 months	34	22				
≥ 87 months	66	13	< 0.001	0.73	0.29 to 1.82	0.501

*CI* confidence interval, *HR* hazard ratio, *MR4.5* molecular response of 4.5-log reduction from baseline, *P P* value, *TKI* tyrosine kinase inhibitor

Loss of MR^4.5^ among patients with MR^4.5^ duration sustained for more than 2 years before discontinuation was 29% (26/89) compared to 82% (9/11) for those with MR^4.5^ sustained for less than 2 years. However, 6 years (72 months) of sustained MR^4.5^ before discontinuation was the optimal cutoff with the most significant difference in the rate of loss of MR^4.5^. Loss of MR^4.5^ occurred in only 7% (4/54) of those with MR^4.5^ sustained for 6 years or more before discontinuation but in 67% (31/46) of those with MR^4.5^ sustained for less than 6 years before discontinuation (*p* < 0.001) (Fig. 1g). Patients with MR^4.5^ duration of 2 to 4 years and 4 to 6 years had MR^4.5^ loss rates of 67% (10/15) and 60% (12/20), respectively. To better understand the probability of loss of MR^4.5^ for those with the longest duration of sustained MR^4.5^, we further grouped them in three cohorts. Patients with MR^4.5^ sustained for 6 to 8 years had a relapse rate of 6% (1/17), while no relapses were documented among patients with MR^4.5^ duration of 8 to 10 years (0/20), and 18% of patients (3/17) relapsed after 10 years of MR^4.5^.

Factors that had no correlation with the probability of loss of MR^4.5^ included discontinuation due to adverse events vs. electively (relapse rate 38% and 32%, respectively; *p* = 0.56) (Fig. 1h), transcript type and Sokal score (Table 2).

Multivariate analysis considering duration of sustained MR^4.5^, frontline TKI vs prior interferon, number of lines of treatment received, and duration of TKI therapy was performed. MR^4.5^ duration of ≥ 72 months before discontinuation (*p* = 0.001; HR = 0.11, 95% CI [0.03–0.41]) was the only significant predictor for durable response (Table 2).

### Factors associated with loss of MMR

We then analyzed the factors associated with loss of MMR. For the purpose of this analysis, the 14 patients who resumed therapy after loss of MR^4.5^ but not MMR (*n* = 12) or without loss of MR^4.5^ (*n* = 2) were not considered to have an “event” (i.e., loss of MMR). The optimal cutoff of MR^4.5^ duration before discontinuation with the most statistical significance for loss of MMR was 25 months. The rate of loss of MMR was 12% (11/89) for those with MR^4.5^ sustained for 25 months or more before discontinuation and 55% (6/11) for patients with MR^4.5^ sustained less than 25 months before discontinuation (*p* = 0.001).

There was a non-statistically significant trend for a lower rate of loss of MMR in patients achieving MR^4.5^ within 17 months compared to patients in whom such response occurred after 17 months (11% and 23%, respectively; *p* = 0.11). There was also a trend for lower rate of loss of MMR in patients who discontinued due to adverse events compared to those who discontinued electively (13% and 22%, respectively; *p* = 0.19). Median duration for TKI therapy was 108 months. There was no statistical difference in the loss of MMR rate between patients who received TKI for more than 108 months compared to less than 108 months (relapse rate 12% vs 22%, respectively; *p* = 0.23). Similarly, the rate of loss of MMR did not differ between patients who received interferon vs those who received frontline TKI (16% vs 18%, respectively; *p* = 0.75). There was also no difference in the rate of loss of MMR between patients who had only received one TKI compared to those who had received two or more TKI by the time they discontinued therapy (15% vs 22%, respectively; *p* = 0.31). There was no difference in loss of MMR rate between patients who received imatinib, dasatinib, or nilotinib as their frontline and only TKI 17%, 18%, and 14%, respectively (*p* = 0.97). Patients receiving imatinib, dasatinib, or nilotinib as their last TKI before discontinuation lost MMR at a rate of 15%, 20%, and 21%, respectively (*p* = 0.75).

Due to the low number of events, only two variables were included in a multivariate analysis and included duration of sustained MR^4.5^ and time to achieve MR^4.5^. MR^4.5^ duration of ≥ 25 months before discontinuation was the only significant predictor for durable response (*p* = 0.003; HR = 0.22, 95% CI [0.08–0.61]).

## Discussion

Patients with CML who are treated with TKI can have a relative survival comparable to that of the general population [27]. However, despite the significant efficacy and overall safety of therapy with TKI, prolonged treatment with these drugs may carry some drawbacks. Patients frequently experience chronic adverse events that are usually low-grade but may impact their overall well-being. In addition, there is the increasing recognition of late adverse events such as arterio-thrombotic events [11] and the possible increased risk of second cancers found in some [28] (but not all [29, 30]) reports. In addition, increasingly important, chronic therapy with TKI generates a financial burden to patients and to society [10]. The pioneering studies of Mahon et al. [15] and Ross et al. [16] demonstrated that TKI discontinuation after achieving a deep molecular response for at least 2 years could be successful in a significant percentage of patients. We here report the largest single-institution experience and the first experience from the US on treatment discontinuation, showing generally similar results as those previously reported.

The initial reports of the Stop Imatinib (STIM) and the CML8 (TWISTER) trials, two multicenter prospective studies initially reported with a median follow-up of 65 and 42 months, respectively, showed an incidence of molecular relapse of 61% and 63%, respectively [15, 16]. Our study shows that among patients with MR^4.5^ at discontinuation, loss of MR^4.5^ occurred in 35 patients (35%) at a median of 4 months. Three late relapses occurred in the STIM study at 19, 20, and 22 months, and no relapses beyond 27 months were observed in TWISTER study [15, 16]. In our study, six patients lost MR^4.5^ 12 months or later after treatment discontinuation (range 16–34 months). This includes one patient who received ponatinib for 15 months with a sustained MR^4.5^ for 9 months before discontinuation and lost MR^4.5^ 16 months after discontinuation.

Important criteria to consider when planning discontinuation of treatment is the duration and depth of response. Most reported and ongoing trials on imatinib, nilotinib, or dasatinib discontinuation require at least 2–3 years of treatment duration and 1–2 years of deep molecular response duration before discontinuation [15–21]. An interim analysis of the EURO-SKI trial reported loss of MMR in 47% of patients treated < 8 years, as compared to 26% treated for > 8 years [17]. In our initial experience, we had reported a lower probability of relapse for patients who sustained complete molecular response of more than 64 months before discontinuation [6]. In this larger experience with considerably more patients and follow-up, we confirm this observation. Our results show that having MR^4.5^ duration of at least 2 and 6 years is associated with 29% and 7% probability of loss of response, respectively. Only four patients lost MR^4.5^ among the 54 with an MR^4.5^ sustained for 6 years or more; three of them also lost MMR and all four regained MR^4.5^ a median of 3 months (range, 2–8) after resuming therapy. These four patients had a sustained MR^4.5^ for 92, 121, 135, and 158 months. The fact that these four patients lost their response despite a very long MR^4.5^ duration and the few but important late relapses mentioned earlier underscore the need for continued monitoring of patients long (and possibly indefinitely) after treatment discontinuation. It also highlights the need for continued follow-up of the treatment discontinuation studies. It is important to emphasize that relapses after stem cell transplant, an option of unquestionable curative potential, can occur more than 15 years after transplant [31]. This suggests that leukemic cells may persist, perhaps in a dormant state, for long periods of time and remain capable of proliferation triggering recurrence of the disease in at least a few instances. The mechanisms of disease control after these two treatment modalities are different; thus, this scenario may or may not replicate after TKI treatment discontinuation and/or may occur at a different rate. Still, continued monitoring appears at the moment a judicious practice for these patients.

Importantly, other studies have suggested that duration of TKI therapy (rather than duration of sustained MR^4.5^) is the critical determinant of the probability of relapse after treatment discontinuation. In a recent update from the STIM trial, Etienne et al. showed that imatinib treatment duration of at least 54 months was associated with lower risk of molecular relapse [32]. Our data showed that patients who received TKI for 87 months or more had lower rate of loss of MR^4.5^ compared to patients with less than 87 months TKI duration but only the duration of MR^4.5^ was statistically significant by multivariate analysis. The low relapse rate seen in patients with sustained MR^4.5^ for 6 years or more is auspicious and, if confirmed in other series, may be translate into the safest guideline for treatment discontinuation.

Initial trials had strict criteria for stopping TKI defined mainly as having undetectable transcript levels by PCR with PCR with 5-log sensitivity (i.e., at least 100,000 copies of control gene in real-time PCR) [15, 16]. Subsequent studies like the French multicenter observational study had more flexible criteria where patients in MR^4.5^ were considered eligible for discontinuation showing a 36% loss of MMR after a median follow-up of 31 months [18]. In our study, although 35% of patients with MR^4.5^ before discontinuation lost MR^4.5^ at a median of 4 months, only 17% lost MMR. This rate was influenced by the fact that some patients were retreated after losing MR^4.5^ and before losing MMR (*n* = 12, 34%). However, six patients who lost MR^4.5^ have not lost MMR despite having no retreatment after a median follow-up of 26 months with two patients spontaneously regaining MR^4.5^. This shows that despite persistence of small detectable levels of leukemic cells, patients can stay in clinical remission. Disease control without treatment after achieving a deep level of response may be attributed to the immune surveillance system that may be able to control the disease when the disease burden is low.

Initial studies reported data on imatinib discontinuation [15, 16, 18]. More recent reports are assessing the outcome after nilotinib or dasatinib discontinuation [16–21]. A recent study on patients who discontinued therapy with nilotinib used as frontline therapy reported that 52% of 190 patients who stopped therapy maintained MMR 48 weeks after stopping [20]. A dasatinib discontinuation trial (DADI trial) showed 52% relapse rate after 20 months median follow-up in 63 patients receiving dasatinib as second line or subsequent therapy who discontinued treatment after maintaining deep molecular response for longer than 1 year [21]. Our data shows that patients receiving imatinib, dasatinib, or nilotinib as their last TKI before discontinuation lost MR^4.5^ at a rate of 23%, 46%, and 43%, respectively, and lost MMR at a rate of 15%, 20%, and 21%. These rates are probably influenced to the same extent the favorable effect of prior interferon, which is mostly seen in the imatinib cohort, and the negative effect of having received prior TKIs which mostly affects the nilotinib and dasatinib cohorts. Also, the median duration of treatment with imatinib, dasatinib, and nilotinib before discontinuation was 121 (range, 11–198), 65 (range, 5–163), and 71 (range, 1–119) months, respectively. When considering only patients receiving either of these drugs as frontline therapy, the loss of MR^4.5^ rate was equalized: 8/23 (35%), 4/13 (31%), and 2/7 (29%), respectively. However, because the sample size for frontline dasatinib and nilotinib is small, these conclusions should be considered preliminary. Still, the rates of relapse after discontinuation appear to be similar for all TKIs. This suggests that the response drives the outcome rather than the specific TKI. This is not surprising considering that none of the TKIs is known to be able to eradicate the earlier CML progenitors in vitro. It also follows other observations where the response drives the outcome rather than the TKI used to achieve such response. For example, for patients achieving early molecular response, the outcome is similar regardless of whether they achieve this response with a second-generation TKI or with imatinib. What is different is the greater likelihood of achieving early molecular response (or in our case, sustained MR^4.5^) with second-generation TKIs than with imatinib.

Our analysis confirms a significant increase in loss of MR^4.5^ rate in patients who were on their second or subsequent line of TKI compared to patients who discontinued their first TKI (*p* = 0.007). We however did not find a difference between patients who discontinued therapy electively versus those who stopped because of adverse events, provided they meet similar criteria for discontinuation. A report by Rea et al. on 60 patients who were on second-generation TKI for 3 years and in MR^4.5^ for 2 years shows that the probability of achieving a treatment-free remission at 48 months was markedly inferior in patients with prior resistance to imatinib compared with those who had imatinib intolerance (18% vs 80%, respectively) [19]. In our analysis, we found no significant difference in the rate of loss of MR^4.5^ after discontinuation in patients who have received more than one TKI that switched to their last TKI due to intolerance (*n* = 25) or resistance (*n* = 6) (48% vs 67%, respectively; *p* = 0.41). This discordant result compared to the recent series from Rea et al. might be only the result of the ample confidence intervals due to the small numbers of patients (60 patients in the French study, 31 in ours). Further studies are needed to define this aspect of treatment discontinuation to determine the safety and optimal circumstances of treatment discontinuation in patients who have experienced failure to prior TKI.

In conclusion, in this experience from a single institution, our results show that TKI treatment discontinuation is safe in most patients when done in the right circumstances and effective in more than half of the patients. Our results suggest that the risk of relapse is minimal for patients who have sustained MR^4.5^ for at least 6 years. If confirmed, this guideline may be used to minimize risks for patients contemplating treatment discontinuation.

## Abbreviations

CART: Classification and regression tree
CCyR: Complete cytogenetic response
CI: Confidence intervals
CML: Chronic myeloid leukemia
HR: Hazard ratio
IRB: Institutional review board
IS: International scale
MMR: Major molecular response
NCCN: National Comprehensive Cancer Network
qPCR: Quantitative real-time polymerase chain reaction
TKI: Tyrosine kinase inhibitor
